# The utilization of antenatal care among rural-to-urban migrant women in Shanghai:a hospital-based cross-sectional study

**DOI:** 10.1186/1471-2458-12-1012

**Published:** 2012-11-21

**Authors:** Qi Zhao, Zhihuan Jennifer Huang, Sijia Yang, Jie Pan, Brian Smith, Biao Xu

**Affiliations:** 1 Department of Epidemiology, School of Public Health, Fudan University, Shanghai 200032, China; 2 Department of International Health, School of Nursing and Health Studies, Georgetown University, Washington, DC, USA

**Keywords:** Antenatal care, Migrant, Pregnancy, Health services utilization, China

## Abstract

**Background:**

Improving utilization of antenatal care is a critical strategy for achieving China’s Millennium Development Goal of decreasing the maternal mortality ratio (MMR). While overall utilization has increased recently in China, an urban vs. rural disparity in access remains. Here we aim to assess utilization of antenatal care in rural-to-urban migrant women and identify its risk and protective factors.

**Methods:**

Migrant women who had been living in Shanghai for more than six months, delivered in one of the two study hospitals between August 2009 and February 2010, and provided written consent were interviewed using a structured questionnaire.

**Results:**

Of 767 women, 90.1% (691) made at least one antenatal care visit, while 49.7% (381) had adequately utilized antenatal care (i.e., made five or more antenatal care visits). Only 19.7% of women visited an antenatal care center during the first trimester (12 weeks). Women between the ages of 25 and 30 and women older than 30 were more likely than younger women to have adequately utilized antenatal care (AOR=2.2 and 1.9, 95%CI=1.4-3.5 and 1.1-3.2, respectively). Women whose husbands held Shanghai residency status (AOR=4.9, 95%CI=2.2-10.9) or who had more than 10 years of education (AOR=1.8, 95%CI=1.2-2.9), previously experienced a miscarriage or abortion (AOR=2.2, 95%CI=1.3-3.8), had higher household income (AOR=1.6, 95%CI=1.0-2.5) were more likely to have adequately utilized antenatal care. Women from high-income households were also more likely to receive antenatal care during the first 12 weeks (AOR=3.5, 95%CI=1.7-5.5).

**Conclusions:**

Many migrant women in Shanghai did not receive adequate antenatal care and initiated antenatal care later than the optimal first 12 weeks of pregnancy. Poor antenatal care utilization was associated with low socioeconomic status, education, and certain demographic factors. Tailored health education for both migrant women and their husbands should be strengthened to improve maternal health. Financing supports should be provided to improve the utilization of antenatal care.

## Background

The maternal mortality ratio (MMR) measures the number of deaths (per 100,000 live births) of women while pregnant or within 42 days of termination of pregnancy from any cause related to pregnancy. The MMR is one of the most important and globally-recognized indicators for measuring the state of a country’s economy, culture, and healthcare system [1].

As a large and low-resource country, China is home to a high number of maternal deaths, accounting for 2% of all maternal deaths worldwide [2]. Twenty years ago, MMR data and information about its influencing factors in China were inaccessible [1]. Since the early 1980s, utilization of maternal healthcare services has grown while antenatal and maternal health indicators have improved [3]. In 1989 the Chinese Ministry of Health established a maternal mortality surveillance system covering the 31 provinces, autonomous regions, and municipalities of the mainland in order to assess changes in the MMR, the main causes of maternal death, and factors influencing the MMR, as well as to provide data for policy-making. Today, China is on track to achieve the Millennium Development Goal of reducing the MMR by 75% from 1990 to 2015. According to a national maternal and infant mortality survey, the MMR had declined from 89/100,000 in 1990 to 31.9/100,000 in 2009 [4,5]. The MMR reduced about 65% within two decades which can be attributed primarily to China’s rapid economic development and improved healthcare services [6]. However, it is obvious that the large disparities in the MMR remained. The MMR varied between rural and urban areas and among different regions in China which may be due to the unbalanced nature of economic development and disparities in access and utilization of healthcare services across regions [7].

Recently a higher MMR among domestic migrant populations compared to permanent residents in the same city has emerged in China [8]. Such a disparity is not surprising as the process of migration can be arduous, particularly for more vulnerable groups like the less educated and women and children. In addition, rural-to-urban migrants in China hold low social status and receive low wages, yet they have also been excluded from benefits that those in the legal workforce receive – such as health insurance – thus exacerbating their vulnerability [9].

Health literature from both developed and developing countries has consistently documented a positive association between reductions in maternal mortality and utilization of maternal healthcare [10-14]. Timely and adequate antenatal care has been found to be associated with reduction negative birth outcomes such as low-birth weight, preterm delivery [15]. Accessing antenatal care in a timely manner enables women to receive information early in their pregnancy concerning the full range of screening tests available [16]. Women who fail to initiate antenatal care during the first trimester may be denied the opportunity to benefit from the full range of early pregnancy screening tests. The new World Health Organization (WHO) antenatal care model recommends a minimum of four visits and provides detailed instructions on the basic components of antenatal care across developed and developing countries [17]. In China, as broadly outlined in the 1994 Law of the People’s Republic of China on Maternal and Infant Health Care, the indicators for antenatal care utilization are: use of antenatal care (i.e., making at least one antenatal visit), initiating antenatal care during the first trimester of pregnancy (i.e., within the optimal first 12 weeks of pregnancy), and making five or more antenatal care visits [4].

As China’s financial and economic center, Shanghai is a popular destination for rural migrants from the surrounding provinces, particularly Anhui and Jiangsu [18]. According to a report of the Shanghai Health Bureau, non-resident pregnant women accounted for 40% of the pregnancies in Shanghai in 2005. It is a shocking fact that among the total 20 reported maternal deaths in Shanghai in 2004, 14 were non-resident migrants. The most obvious reason for the high MMR among non-resident women in Shanghai is lack of equitable access to maternal care [19]. The migrant women are not covered by the social medical insurance for Shanghai residents. They are required to pay out of pocket for all health care, including legally obligated hospital delivery and other essential maternal care. The fee for a normal vaginal delivery is 2500CNY (1USD=7CNY) while the fee for a cesarean delivery is 5000CNY. In relation to the average monthly income of 800CNY/month for the migrants, the delivery fees represent a heavy burden. In order to improve accessibility of the maternal health care service, the Shanghai government launched a “low-price delivery service” project in August 2004 to make maternal health services accessible to non-resident pregnant women, under the principle of “sharing payment among government, hospital and individual”. The maximum fee for a vaginal delivery under the scheme is 800CNY,covering the expenditure on necessary items, including delivery fee, oxytocin injection after delivery, physical examination of the newborn baby, HBV and BCG vaccinations, follow-up nursing within 24-hours of delivery and necessary treatment. The woman should be discharged within 24 hours after delivery. The criteria for accessing the project varied in different districts. In the framework of the project it is recommended that non-resident pregnant women have at least three antenatal check-ups (ANCs) with an upper limit payment of 150 CNY. The designated low-price delivery hospitals are obliged to refer pregnant women with severe complications to secondary or tertiary hospitals. But in 2008, after 4 years implementation of low price delivery service policy, Gao and her colleagues still found that only 38.3% of rural-to-urban migrant women received an obstetric exam during the optimal first trimester of pregnancy, and only 36.6% received five or more antenatal exams during pregnancy [20]. Liu and her colleagues investigated 600 floating (migrant) women aged from 15 to 49 in Pudong new area and found that the proportion of prenatal examination was 55.8% [21].

The number of non-residents (i.e., those lacking *hukou*, or residency status for a particular city) living in Shanghai has increased greatly in the past 25 years, from 600,000 in 1984 to 5,410,000 in 2009, now accounting for more than one-fourth of Shanghai’s total population [22]. Sixty three percent of the rural to urban migrants are at the age of 20-39 [23], most of them are unskilled and have limited education from primary schools and/or junior high schools. The large-scale migration of women in reproductive age is having a huge impact on maternal services in Shanghai. According to the Shanghai health report, the MMR for shanghai resident and migrant women reduced 77% and 75% respectively. However, there are still huge gap between shanghai residents and migrant while the overall MMR were comparable to levels in developed countries. The MMR for non-resident migrants in Shanghai was13.54/100,000, about 2.5 times higher than the MMR for shanghai residents, 5.30/100,000 in 2010. The challenge we faced in Shanghai is to improve maternal health for the rural migrant women. [24]. This study aims to address this gap by assessing utilization of antenatal care among these migrant women living in urban areas of Shanghai where covered by the low price delivery service policy. Specifically, it aims to identify associations of demographic characteristics, socioeconomic status, pregnancy history, and awareness of and personal opinion concerning antenatal care with utilization of prenatal care. This study also aims to provide evidence-based data for future policies designed to improve maternal healthcare among rural-to-urban migrant women.

## Methods

### Study sites

This cross-sectional study was carried out in the obstetrical departments of two general hospitals in Shanghai: Changning District Center Hospital (CNH) and Punan Hospital (PNH). These hospitals are located in the Changning and Pudong suburban districts, respectively, and both hospitals offered low price delivery service for migrant women. In 2008 the number of non-Shanghai residents who had migrated from rural areas to these two districts was 130,200 and 1,273,300, respectively, accounting for 17.5% and 39.6% of the districts’ total populations. In Changning District the number of births in 2008 among local residents was 3,758 with a birth rate of 6.14 per thousand compared to 1,123 births and 8.63 per thousand among non-Shanghai residents. In Pudong District, the number of births in 2008 among local residents was 15,962 with a birth rate of 8.28 per thousand, compared to 14,048 births and 11.03 per thousand among non-Shanghai residents [25].

### Study subjects

Study subjects were women who had migrated from rural areas of China to Shanghai (despite lacking Shanghai residency status, or *hukou*) and gave birth in one of the two aforementioned hospitals between August 2009 and February 2010. With the assumption of the proportion of having more than 4 antenatal care visits as 50% and a precision of ±5% for the 95% confidence interval (α=0.05, *d*=0.05p), the estimated sample size was 385. Finally a sample of 400 eligible women in each district was expected.

### Data collection

Women were recruited somewhat differently within the hospitals due to differing in health management systems. At the CNH site all subjects were recruited from the prenatal department within 42 days after delivery, while at the PNH site subjects were recruited in the obstetrical department within 10 days after delivery. A questionnaire was designed to collect data concerning women’ demographic and migration information (age, ethnicity, hometown, duration of stay in Shanghai, and husband’s residency status), socioeconomic status (years of education, occupation and husband’s occupation, and self-reported household income), previous and current pregnancy details, and awareness of and personal opinion concerning antenatal care. Face-to-face interviews based on the questionnaire were used to collect data by the medical students from Fudan University. A pilot study was conducted at the CNH site during the first month of the study in order to assess the questionnaire and investigation methods. Throughout the study period, 10% of women who participated in an interview were randomly selected to be further interviewed in order to assess quality of data.

### Data analysis

All data were double-entered using EpiData 3.1.0. The database was analyzed using SPSS for Windows Version 11.0 (SPSS, Chicago, IL, USA). Utilization of antenatal care was defined as having made at least one antenatal visit before delivery. Timely utilization of antenatal care was defined as making the initial prenatal care visit within the first trimester of pregnancy (i.e., during the optimal first 12 weeks of pregnancy). Adequate utilization of antenatal care was defined as having made five or more antenatal care visits during pregnancy.

Descriptive indices such as proportion, mean (and median for skewed distribution) and quartiles were used to assess the general characteristics of the pregnant women, their utilization of antenatal care, and their awareness of and personal opinion concerning antenatal care. When measuring the income levels, women’s household income levels were grouped into three strata based on the tertile of annual income, which were applied from the poorest 1/3 (low) to the richest 1/3 (high). Bivariate and multivariate logistic regressions were used to analyze the overall and independent associations of socioeconomic characteristics and other factors with utilization of prenatal care. Multivariate analysis with logistic regression model was applied to analyze the influences of socioeconomic and non-socioeconomic factors on the utilization of antenatal care visit. All the dichotomous variables were analyzed in terms of the events happening, i.e. the comparison was 1 to 0. All the group variables were analyzed with dummy variable. Each dummy variable was compared with the first group. Table 1 was the definition of variables used in the analysis.

**Table 1:** Definition of variables used in the analysis

**Variables**	**Value**
Dependent variables	
Adequate utilization	0: having less than 5 visits 1: having adequate visits
Timely utilization	0: later than 12 weeks 1: having first visit during 12 weeks
Independent variables	
Age group	1:<25 years old, 2:25–30; 3>30 years
Husband’s residency status	1: Shanghai; 0 Other
Women’s education	0: less than 10 years; 1: 10-
Employment status	1: Unemployed throughout; 2: Before & during pregnancy; 3: Before pregnancy only;
Health insurance	0: without; 1: with
Husband’s years of education	0: less than 10 years; 1: 10-
Household income (by tertile)	1: Low; 2: Middle; 3: High;
Pregnancy and delivery history	1: Previously delivered (mutlipara); 2: Previously pregnant but did not deliver; 3: First pregnancy

### Ethic consideration

Informed consents were sought from all the participants after giving a description of the study prior to the interview. Approval for this study was obtained from the Ethics Committee of the School of Public Health, Fudan University.

## Results

### General information

A total of 775 women were recruited into this cross-sectional study. Every woman recruited agreed to participate and provided consent. Eight women (1%) were excluded from our analysis, as three held Shanghai residency status and five did not provide any information on antenatal care visits. The two hospitals were sampled equally with 380 (49.5%) women from Changning and 387 (50.5%) women from Pudong. The demographic and geographic information of investigated women were comparable between these two hospitals.

Of the 767 eligible women, 39.0% and 11.9% were from neighboring Anhui and Jiangsu Provinces, respectively. The remaining 49.1% were from one of China’s 19 other provinces. Table 2 showed that the mean age of women was 26 ± 5 years old. The median duration of stay in Shanghai was 4.45 years. The majority of women (55.4%) and their husbands (55.6%) had seven to 10 years of education. Only 15.5% of women worked both before and during pregnancy, while 50.1% stopped working after becoming pregnant. The median incomes of each stratum were 5,000, 10,000, and 22,500 RMB Chinese Yuan. This was the first pregnancy for 35.7% (294) of women, while 45.1% (346) had previously given birth and 19.2% (147) had either miscarried or undergone an abortion. Only 4.1% (31) of women did not know that antenatal care was required, while 92.4% believed that antenatal care was a necessity.

**Table 2:** General Information of study subjects (n=767)

		**No.**	**%**
*Demographic information*			
Age (years)*		26±5	
Duration of stay in Shanghai (years) **		4 (2–7)	
Husband’s residency status	Shanghai	74	9.6
	Other	693	90.4
		*Socioeconomic information*			
Women’s years of education	More than 10 years	239	31.4	
	7-10 years	422	55.4	
	Less than 7 years	93	12.2	
	Illiterate	8	1.0	
Employment status	Before & during pregnancy	119	15.5	
	Before pregnancy only	384	50.1	
	Unemployed throughout	264	34.4	
Health insurance status	Rural cooperation insurance	370	49.8	
	Other ^a^	86	12.6	
	None	287	38.6	
Husband’s years of education	More than 10 years	283	39.1	
	7-10 years	402	55.6	
	Less than 7 years	36	5.0	
	Illiterate	2	0.3	
Household income (CNY)**	Low	269	5000(3333–6250)	
	Middle	219	10000(9000–12000)	
	High	243	22500(18000–33333)	
Pregnancy and delivery history				
First pregnancy		274	35.7	
Previously pregnant but did not deliver		147	19.2	
Previously delivered		346	45.1	
Yes		730	95.9	
No		31	4.1	
^a^Personal opinion of antenatal care				
Necessary		705	92.4	
Unnecessary		58	7.6	

* Mean±Std.

** Median (25% - 75%).

^a^ Including Shanghai urban resident basic medical insurance, Shanghai rural cooperative medical scheme, and commercial health insurance.

### Utilization of antenatal care

Seventy-six women (9.9%) never made an antenatal care visit before delivery, while 90.1% (691) of participants made at least one visit. Among the women having made antenatal care visit, the average number of visits was 5.35±3.05. The median number of visits was four with an inter-quartile range of two to eight visits. A total of 49.7% (381) of women had adequately utilized antenatal care (i.e., made at least five antenatal care visits before giving birth). Among the 386 women having less than five antenatal visits, 78 (20.2%) reported that they did not have enough money for antenatal care visits, 77(19.9%) did not think they should have so many visits, 54 (14.2%) had no time for antenatal care visit, and 53 (13.7%) did not know how many visits they should have.

The mean week of pregnancy at time of first antenatal care visit was 23.15±5.7 weeks, and varied from three to 40 weeks. Only 19.7% (130) of women visited an antenatal care center during the optimal first 12 weeks.

### Factors associated with adequate utilization of antenatal care

Table 3 showed the factors associated with *adequate* utilization of antenatal care. Multivariate logistic regression showed that, compared with younger women, women between the ages of 25 and 30 (AOR=2.2, 95%CI=1.4-3.5) and women older than 30 (AOR=1.9, 95%CI=1.1-3.2) were more likely to have adequately utilized antenatal care. Women who had more than 10 years of education (AOR=1.8, 95%CI=1.2-2.9) or whose husbands held Shanghai residency status (AOR=4.9, 95%CI=2.2-10.9) were more likely to receive adequate antenatal care. We also found that women who were previously pregnant but did not deliver (AOR=2.2, 95%CI=1.3-3.8), had a high household income (AOR=1.6, 95%CI=1.0-2.5) were more likely to have made five or more antenatal care visits, after adjusting for duration of stay in Shanghai, health insurance status, and employment status during pregnancy.

**Table 3:** Factors Associated with Adequate Utilization of Antenatal Care

		**No.**	**%**	**COR(95%CI)**	**AOR(95%CI)**
Age	<25 years	150	44.3	1	1
	25-30 years	138	61.6	1.0(0.7-1.4)	**2.2(1.4-3.5)**
	>30 years	82	45.3	1.9(1.3-2.9)	**1.9(1.1-3.2)**
Husband’s residency status (*hukou*)	Other	317	45.7	1	1
	Shanghai	64	86.5	7.6(3.8-15.0)	**4.9(2.2-10.9)**
Years of education ^a^	<10 years	209	40.0	1	1
	>=10 years	170	71.1	3.7(2.6-5.1)	**1.8(1.2-2.9)**
Employment status	Unemployed throughout	112	42.4	1	1
	Before only	194	50.5	1.4(1.0-1.9)	1.3(0.9-2.0)
	Before and during	75	63	2.3(1.5-3.6)	1.1(0.7-2.1)
Health insurance	without	134	46.7	1	1
	with	236	51.8	1.2(0.9-1.6)	1.0(0.7-1.5)
Husband’s years of education	<10years	167	38	1	1
	>=10 years	198	70	3.8(2.8-5.2)	**1.9(1.3-2.9)**
Household income	Low	105	39	1	1
	Middle	105	48	1.4(1.0-2.1)	1.3(0.9-2.0)
	High	156	64.2	2.8(2.0-4.0)	1.6(1.0-2.5)
Pregnancy and delivery history	Previously delivered	142	41	1	1
	Previously pregnant, did not deliver	96	65.3	2.7(1.8-4.0)	**2.2(1.3-3.8)**
	First pregnancy	143	52.2	1.6(1.1-2.2)	1.5(0.9-2.4)

*COR* crude odds ratio.

*AOR* adjusted odds ratio.

### Factors associated with timely utilization of antenatal care

Table 4 outlined the factors associated with *early* utilization of antenatal care. We found that compared to their lower-income counterparts, women from high-income households were more likely to have received antenatal care during the optimal first 12 weeks of pregnancy (AOR=3.5, 95%CI=1.7-5.5).

**Table 4:** Factors Associated with Early Utilization of Antenatal Care

		**No.**	**%**	**COR(95%CI)**	**AOR(95%CI)**
Age	<25 years	63	21.7	1	1
	25-30 years	30	15.5	0.7(0.4-1.1)	0.7(0.4-1.2)
	>30 years	33	21	1.0(0.6-1.5)	1.3(0.6-2.5)
Husband’s residency status (*hukou*)	Other	111	18.9	1	1
	Shanghai	19	26	1.5(0.9-2.7)	1.8(1.0-3.6)
Years of education ^a^	<10 years	89	20.3	1	1
	>=10 years	40	18.4	0.9(0.6-1.3)	0.8(0.4-1.3)
Employment status before and during pregnancy	None	49	22.2	1	1
	Before only	66	19.9	0.9(0.6-1.3)	0.8(0.5-1.3)
	Before and during	15	13.9	0.6(0.3-1.1)	0.5(0.2-1.0)
Health insurance status	Without	41	17.5	1	1
	With	83	20.5	1.2(0.8-1.8)	1.3(0.8-2.1)
Husband’s years of education	<10 years	75	20.4	1	1
	>=10 years	50	19.5	0.9(0.6-1.4)	0.8(05–1.4)
Household income	Low	33	14.7	1	1
	Middle	42	21.8	1.6(1.0-2.7)	**1.9(1.1-3.4)**
	High	54	25.2	2.0(1.2-3.2)	**3.5(1.7-5.5)**
Pregnancy and delivery history	Previously delivered	54	18.4	1	1
	Previously pregnant, did not deliver	19	14.2	0.7(0.4-1.3)	0.7(0.4-1.4)
	First pregnancy	57	24.5	1.4(1.0-2.2)	1.7(0.9-3.0)

*COR* crude odds ratio.

*AOR* adjusted odds ratio.

## Discussion

Our study found that rates of antenatal care utilization among migrants in Shanghai were very high: 90.1%. However, the number of antenatal care visits tended to be inadequate and the initial visit usually occurred after the first trimester. Just under half of women (49.7%) made five or more antenatal care visits before delivery, and only 19.7% (130) of women visited an antenatal care center during the first trimester. Several factors, including husband’s residency status, education level of the woman and her husband, history of pregnancy and delivery, household income and age, were associated with the adequate utilization of antenatal care while only household income and age of pregnancy women were significantly associated with early utilization of antenatal care.

### Lack of timely and adequate utilization

As an important component of primary healthcare, adequate antenatal care is generally considered essential for reducing adverse birth outcomes [15]. Adequate antenatal care has also been found to be an indicator of utilization of future child healthcare, including adequate number of well-child visits and up-to-date immunization status [26,27]. Many studies have found that coverage of antenatal care in middle- or low-income countries is increasing, though the number of antenatal care visits was inadequate [28-31]. Similar to our study findings, a survey conducted by the China CDC in Beijing and Hangzhou in 2007 reported that 51% of migrant women initiated antenatal care during the optimal first trimester and 59.5% of women made at least five antenatal visits, both of which were lower than rates for women who held urban residency status [32]. Chen found that most migrant women in the city of Ningbo had never initiated antenatal care, which led to a high MMR [33]. A 2009 study conducted in eastern Sudan found that 90% of women had made at least one antenatal care visit, though only 11% of women made at least four antenatal care visits [28,34].

Timely and adequate prenatal care has been found to be associated with a reduction in low birth weights [15]. Initiating prenatal care during the first trimester can also reduce poor pregnancy outcomes [35,36]. Women who access antenatal care early in their pregnancy are able to receive timely information concerning the full range of antenatal screening tests available [16], while women who initiate antenatal care after the first trimester may be denied the opportunity to benefit from these same screening tests. Mrisho et al. found that the most common reason cited for delaying initiation of antenatal care in rural Tanzania was to avoid having to make several visits to the clinic [37]. A 2009 cross-sectional study in 10 Chinese provinces found that the coverage rate for antenatal care was 95.7%, 50.8% of women received at least five prenatal exams and 25.9% made their initial visit during the optimal first 12 weeks of pregnancy [38]. The low rate (19.7%) of timely utilization of antenatal care in our study was consistent with other studies, which suggest although China has experienced a dramatic development during last 30 years, the antenatal care for rural to urban migrant women is still not satisfactory.

### Factors associated with utilization of antenatal care

Since inadequate antenatal care is associated with worse pregnancy outcomes [39], it is vital for health policymakers to better understand the factors influencing proper and prompt utilization of antenatal care [40]. In this study, it was found that older, better educated women, and women who had previous pregnancies but did not deliver (miscarriage or abortion) were more likely to receive adequate antenatal care. Except for women having better household income, almost all the women had delayed initiation of antenatal care utilization.

Beeckman et al. found that women who were better educated, had higher incomes, or were primiparous (i.e., had only one child) made more antenatal care visits than women who were less educated, had lower incomes, or were multiparous [40]. Friedman et al. found that the primary reasons why women had not received antenatal care were substance use, denial of pregnancy, financial limitations, concealed pregnancy, and having previously delivered [41]. Studies in China have found that inadequate utilization of antenatal care among rural-to-urban migrants is attributable to factors such as financial difficulties, lack of knowledge concerning prenatal care, and low levels of education [42-45]. Ye et al. found that education, marital status, family income, employment status, and duration of stay in the city were the main influencing factors on initiating antenatal care among migrant women in Shenzhou [45]. Liu et al. found that among migrant women in Beijing, Guangzhou, Chengdu and Shanghai, the education levels of women and their husbands as well as family income were the main influencing factors on antenatal care and maternal healthcare utilization [43]. Long et al. reported that women in western China who were less educated, from minority groups, or high parity were less likely to receive antenatal and delivery care [4].

Higher levels of education tend to positively affect health-seeking behaviors, and education may increase a woman’s control over her pregnancy [46]. Education may help to expose women to more health education messages and campaigns, enabling them to recognize danger signs and complications and take appropriate action. Chowdhury et al. found that the odds ratios for use of services among women who had no education or only a primary education were 0.54 and 0.63, respectively, compared to those with at least a secondary education. Similarly, our study found that better educated women received antenatal care more frequently. These women might have greater opportunities to receive health information and pay more attention to maternal healthcare. We found that women who believed that antenatal care was necessary were more likely than those who did not to receive antenatal care and to do so frequently. Hueston et al. found that lower levels of education and prior pregnancy experience were associated with younger teenagers in the US receiving delayed care [47]. A study examining initiation of and barriers to antenatal care among low-income women in San Antonio, Texas found that women who were less educated or living alone, or had not planned their pregnancies were more likely to delay initiating antenatal care [35].

An association of multiparity with low utilization of health services has been reported in studies from Turkey, Indonesia, and Brazil [48-50]. High parity women might tend to rely on their experiences from previous pregnancies and not feel the need for antenatal care exams. Due to their greater level of experience, these women might feel more confident during pregnancy and consider antenatal care to be less important [34]. Some women might not visit an antenatal care service provider due to the responsibilities they already have for their other children or due to negative perceptions developed during previous pregnancies [17,50,51]. However, in our study we only found that the women who had previous pregnancies (miscarriage or abortion history were more likely to receive adequate antenatal care while there was no significant difference between women in their first pregnancy and women who had previously delivered. It is understandable that the experiences of pregnancy failure might have contributed to the improved awareness of antenatal care [52]. This might also be a result of limited number of women in this study who had previous experiences of delivery. Higher parity women and teenagers who were not expecting to become pregnant tend to utilize less antenatal care in developing countries [34].

Financial difficulties have been considered as an important barrier to antenatal care for migrant women. Schillaci et al. found that women living in lower-income areas of the US state of New Mexico received lower levels of antenatal care [15]. A study in rural Tanzania found that women cited lack of money as a reason of delaying antenatal care [37]. Our study found, 20.2% of the women who had inadequate antenatal care reported they did not have enough money for antenatal care visits. Results from multivariate analysis also showed a significant association between income and timely utilization of antenatal care. We have also data from our study subjects to understand how heavy the economic burden of antenatal care and hospital delivery in Shanghai, which will be published in another paper.

### Limitations

This is a hospital-based study, which limits the generalizability and application of its findings. The women might be representative of rural-to-urban migrant women who deliver in hospitals, but not of those migrants who return to their hometowns for delivery or give birth outside the legal maternal healthcare facilities. The cross-sectional study design also limits our ability to find causes of underutilization. During the investigation, the investigated women had to recall previous antenatal care visits and the date, expenses for each visit. So the recall bias might happen. We found some women could not remember the exactly date of each visit, which may be also contribute to the misclassification while analyzing the timely utilization. Therefore, to avoid the bias, women were required to present their medical record, invoices or related vouchers to the investigators.

## Conclusions

This study found only 49.7% of the rural to urban migrant women received adequate antenatal care, while the majority initiated antenatal care after the optimal first 12 weeks of pregnancy. Antenatal care utilization was associated with women’s characteristics such as age, education and pregnancy history, levels of education and residency status of the husbands, and household income. Findings from this study suggest that as the vulnerable population in urban cities, both the rural migrant women and their husbands should be targeted for tailored health education on maternal health, especially the importance and necessity of adequate antenatal care. Financing supports for antenatal care should be another effective way to encourage migrant women having adequate and timely antenatal care utilization. More investigations of the health services needs of pregnant rural-to-urban migrant women should be conducted in the future.

## Competing interests

The authors declare that they have no competing interests.

## Authors’ contributions

Q. Zhao and B. Xu conceptualized the study and drafted the article. Z.J. Huang contributed to data analysis and article editing. S. Yang and J. Pan contributed to data collection. B. Smith contributed to article drafting and editing. All authors read and approved the final manuscript.

## Pre-publication history

The pre-publication history for this paper can be accessed here:

http://www.biomedcentral.com/1471-2458/12/1012/prepub
